# Effects of repeated tuberculin skin testings on immune responses in experimental monkeys

**DOI:** 10.1186/s40781-014-0032-2

**Published:** 2014-12-10

**Authors:** Fangui Min, Jing Wang, Wen Yuan, Huiwen Kuang, Weibo Zhao

**Affiliations:** Guangdong Laboratory Animals Monitoring Institute, Guangzhou, 510663 PR China; Guangdong Provincial Key Laboratory of Laboratory Animals, Guangzhou, 510663 PR China

**Keywords:** Tuberculin skin testing, Tuberculosis, Monkeys, Immune responses

## Abstract

Though many alternative methods to tuberculin skin testing (TST) have been established and evaluated in recent years, sensitivities and specificities of most methods could not meet the requirements of golden standards. In this study, we sought to identify whether repeated TSTs could affect the immune responses in experimental monkeys. Nine natural tuberculosis (TB) monkeys receiving repeated TSTs biweekly were used to demonstrate the effect on TST responsiveness. Two healthy monkeys were administrated with repeated TSTs to analyze the immune response profiling. Intrapalpebral reactions in TB infections gradually weakened or presented intermittent positive reactions. The leukocyte counts, cytokine responses, and antibody responses to all antigens except Old tuberculin (OT) and MPT64L showed no specific changes for TB in healthy monkeys. Positive antibody responses to OT and MPT64L emerged during the first half experimental period, which may cause by their cross-reactivity with mycobacterial species. Results showed that repeated TSTs had no significant effects on immune responses in healthy monkeys but a progressive reduction in TST responsiveness in TB infections.

## Background

Primate tuberculosis (TB) is a zoonotic disease caused primarily by the bacterial pathogen *Mycobacterium tuberculosis* or, less commonly, *M. bovis*. Though most nonhuman primate species are susceptible to TB infection, Old World monkeys are considered more susceptible than New World monkeys [1,2]. The commonly used rhesus (*Macaca mulatta*) and cynomolgous (*Macaca fasicularis*) monkeys in researches belong to Old World monkeys. So the TB surveillance in experimental monkeys becomes a severe challenge.

Diagnosis of primate TB is difficult for the nonspecific clinical features and difficulty in interpreting chest radiographs. Additionally, the clinical features and chest radiographs do not give conclusive evidence for the disease. The golden standard for human TB diagnosis based on bacterial culture of clinical specimens is often not possible in experimental monkeys due to difficulty in collection of sputum specimens. Despite significant improvement in technology and the availability of *M. tuberculosis* genomic sequence data has facilitated the development of newer diagnostic techniques of TB, most of them are still under evaluation or remain limitations [3]. The palpebral tuberculin skin test (TST) has been the mainstay of TB diagnosis in nonhuman primates and the only approved method for TB screening of animals in primary import quarantine. Since the TST is an in vivo test detecting the delayed-type hypersensitivity to tuberculin antigens, the administration of mycobacterial derived antigens may influence the immune response and subsequent immunodiagnostic tests. These were demonstrated in cattle naturally infected with *M. bovis* [4], the repeat short-interval skin-testing could affect not only the skin-test responsiveness but also gamma interferon test.

In present study, we performed a longitudinal investigation to make clear whether repeated TSTs could affect TST responsiveness in TB infections, leukocyte counts, serum cytokines and antibody responses in health monkeys. Results have demonstrated that the repeated TST shows no effect on immune response in TB-negative monkeys but a progressive reduction in TST responsiveness in TB infections.

## Methods

### Animals

Nine TB-positive rhesus monkeys with at least one time of TST-positive reaction by routine quarantines and two healthy monkeys (06-1885R and 06-1891R) aged 3–4 years were used in this study. The healthy monkeys were tested negative for monkey B virus, simian immunodeficiency virus (SIV) and simian T-cell leukemia virus 1 (STLV-1) by ELISA and simian retrovirus (SRV) by immunofluorescence. Animal work was conducted in a negative air pressure facility.

Animal use protocols were reviewed and approved by the Institutional Animal Care and Use Committee of Guangdong Laboratory Animal Monitoring Institute in accordance with the *Guide for the Care and Use of Laboratory Animals* [5].

### Study design

At the interval of 2 weeks, all monkeys were anesthetized intramuscularly with ketamine in combination with xylazine for administration of TSTs and blood samples collection.

### TST procedures

Two kinds of tuberculin, Old tuberculin (OT) and purified protein derivative (PPD), were used in TSTs. OT was from *Synbiotics Corp*. and PPD was from *Harbin Pharmaceutical Group Bio-vaccine Co*. In this study, intradermal palpebral skin testing was performed using 0.1 mL of OT in right palpebra and 0.1 mL of PPD in left palpebra respectively biweekly. TST results were graded at 24, 48, and 72 h using the standard 1 to 5 scoring system [6], and in this study, palpebral reactions of grade 0 ~ 2 were considered negative, those of grade 2 ~ 3 were suspect, and those graded ≥3 were considered positive.

### Total leukocyte and leukocyte population counts

For total leukocyte and leukocyte population counts, about 0.5 ml of heparinized blood samples were assayed on automated hematology analyzer (Sysmex XT-2000iv, Japan).

### Serum antibody detection

Serum antibody responses to 10 *M. tuberculosis* purified recombinant antigens (Ag85b, CFP10, ESAT-6, CFP10-ESAT-6, 38 kDa, 14 kDa, MPT64L, 16 kDa, TB16.3, and U1 protein), PPD, and OT were detected by ELISA as previous report [7].

### Serum cytokine detection

Serum cytokines (TNF-α, IL-8, IL-12/23p40, IFN-γ, IL-2, IL-4, IL-6, and IL-10) were measured by ELISA as previous report [8]. The kits for IFN-γ, IL-2, IL-4, IL-6, and IL-12/23p40 assays were purchased from Mabtech Inc. (Ohio, USA) and for IL-8, IL-10, TNF-α from Bender Medsystems, Inc. (California, USA).

## Results

### Repeated TSTs showed intermittent positive reaction in TB infections

**Figure 1:**
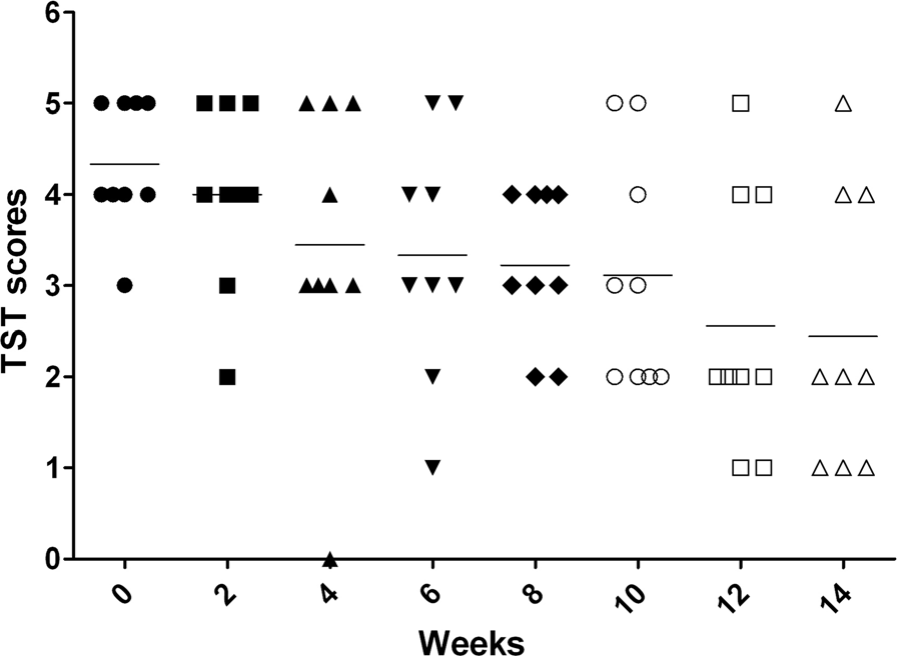
**Results of repeated TSTs.** After repeated TSTs, the scores got weakened gradually in TB infections.

Among nine TB-positive monkeys, eight monkeys were strongly positive (graded 4 ~ 5) at week 0 and one monkey were scored 3. Afterward, intrapalpebral reactions weakened gradually, one monkey (scored 3 at week 0) gave intermittent positive results. At week 12 (the seventh TST) only three monkeys showed positive intrapalpebral reactions (Figure 1). There were no intrapalpebral reactions for the two healthy monkeys.

### Changes in total leukocyte and leukocyte populations

**Figure 2:**
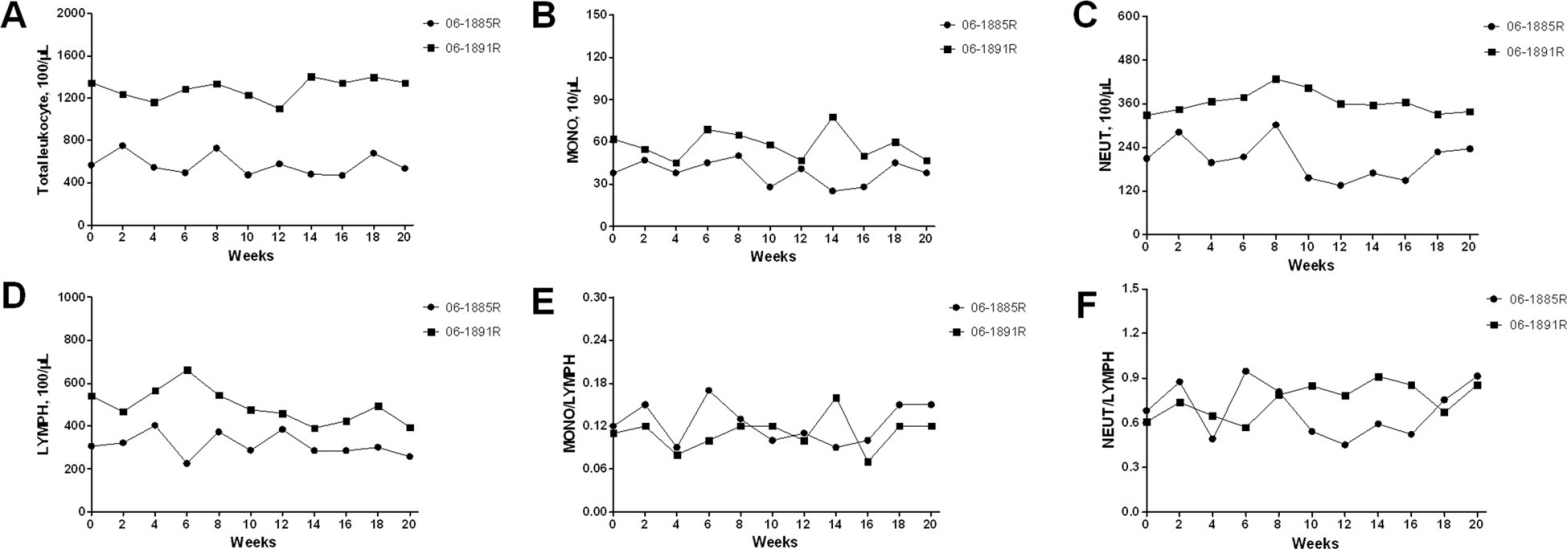
**Results of leukocyte counts. A** **~D** showed the dynamic changes of total leukocyte count, monocytes count, neutrophiles count, and lymphocytes count respectively. **E** and **F** showed Monocyte-Lymphocyte ratio and neutrophile-lymphocyte ratio. There were no significant changes caused by repeated TSTs in leukocyte counts.

There were no significant changes in total leukocyte count and leukocyte population counts including monocytes, neutrophiles, and lymphocytes during the TSTs performed period (Figure 2A~D). The values of monkey 06-1891R were higher than those of monkey 06-1885R. Monocyte-Lymphocyte ratio and neutrophile-lymphocyte ratio, calculated from the numbers of monocytes, neutrophiles, and lymphocytes, showed no obvious elevations (Figure 2E~F).

### Changes in serum cytokines responses

**Figure 3:**
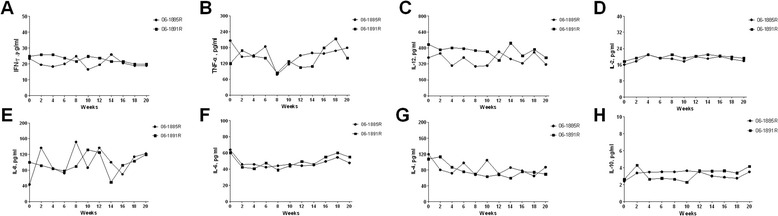
**Results of cytokine responses.** **A~F** showed the dynamic changes of six pro-inflammatory cytokines (TNF-α, IL-12/23p40, IFN-γ, IL-2, IL-6, and IL-8). **G~H** showed those of two anti-inflammatory cytokines (IL-4 and IL-10). No specific changes caused by repeated TSTs were observed.

In this study, six serum pro-inflammatory cytokines and two serum anti-inflammatory cytokines were detected. They were TNF-α, IL-12/23p40, IFN-γ, IL-2, IL-6, IL-8, IL-4 and IL-10. Animals kept baseline all the time for most cytokines. Though there were multipeak kinetics under low levels in some cytokines, no specific changes for TB were observed during TSTs administration course (Figure 3).

### Changes in serum antibody responses

**Figure 4:**
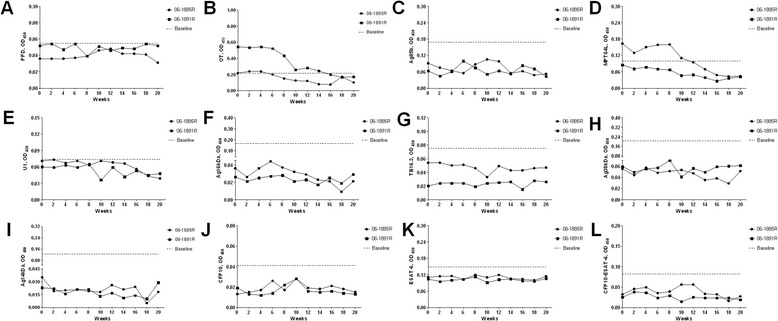
**Results of antibody responses.** Serum antibody responses to OT **(B)** and MPT64L **(D)** showed positive reactions after receiving repeated TSTs, and no specific changes were found in antibody responses to other antigens **(A, C, E~L)**.

Sequential serum samples were tested against selected antigens. Data obtained for all animals in every antigen are present in Figure 4. The baselines were calculated by average plus 3SD according to our previous data [7]. No positive antibody reactions against those antigens except OT and MPT64L were found during TST-treated period. The antibody responses characterizations of OT and MPT64L were similar to each other, moving from positive to negative reactions during the experiment period.

## Discussion

Although the TST in nonhuman primates has many limitations to sensitivity and specificity, it is still kept as the mainstay of tuberculosis screening and antemortem diagnosis. In this study, we tried to make clear whether repeated TSTs in rhesus monkeys affect immune responses, including TST-reaction, total leukocyte and leukocyte populations, serum cytokines responses and antibody responses.

Repeated TSTs in natural TB-infections showed gradually weakened intrapalpebral reactions and intermittent positive results. Our study once again demonstrated the chief limitations of TST in documents. As we previously suggested [8], keeping long enough intervals of TSTs is important in animals’ routine quarantine to draw optimal results, such as quarter quarantine and annual quarantine.

Primary or secondary TB infections were always accompanied by elevated total leukocyte count and leukocyte population counts and a peak in monocyte-lymphocyte ratio and neutrophile-lymphocyte ratio [8,9]. In our study, no significant changes caused by repeated TSTs in healthy monkeys were observed in total leukocyte count and leukocyte population counts. For TB infection, serum cytokines were always induced or suppressed. There were also no specific changes for TB infection in serum cytokines of healthy monkeys. Whether repeated TSTs would affect TB serodiagnostic efficacy still remained open. Now the answer could be obtained from this study. Among 12 selected antigens, only the antibody responses to OT and MPT64L presented periodic positive reactions in healthy monkeys, which may be caused by their non-specificity and cross-reactivity with mycobacterial species [10,11]. Our results showed that repeated TSTs in healthy monkeys could not induce antibody responses and affect TB seradiagnostics.

## Conclusions

In conclusion, repeated TSTs may induce immune tolerance for TST reaction in TB infections, but no effects in leukocyte counts, serum cytokines, and antibody responses in healthy monkeys. Results indicate that the combinatorial use of TST and TB-specific antibody tests may become the overall effectiveness of TB surveillance programs for nonhuman primates.
